# Structure-Driven Performance Enhancement in Palladium–Graphene Oxide Catalysts for Electrochemical Hydrogen Evolution

**DOI:** 10.3390/ma17215296

**Published:** 2024-10-31

**Authors:** Krishnamoorthy Sathiyan, Ce Gao, Toru Wada, Poulami Mukherjee, Kalaivani Seenivasan, Toshiaki Taniike

**Affiliations:** Graduate School of Advanced Science and Technology, Japan Advanced Institute of Science and Technology, 1-1 Asahidai, Nomi 923-1292, Ishikawa, Japan; s2220015@jaist.ac.jp (C.G.); toruwada@jaist.ac.jp (T.W.); poulami@jaist.ac.jp (P.M.); kalaivan@jaist.ac.jp (K.S.)

**Keywords:** graphene oxide, functional groups, Pd nanoparticle, electrocatalysts, hydrogen evolution reaction

## Abstract

Graphene oxide (GO) has recently gained significant attention in electrocatalysis as a promising electrode material owing to its unique physiochemical properties such as enhanced electron transfers due to a conjugated π-electron system, high surface area, and stable support for loading electroactive species, including metal nanoparticles. However, only a few studies have been directed toward the structural characteristics of GO, elaborating on the roles of oxygen-containing functional groups, the presence of defects, interlayer spacing between the layered structure, and nonuniformity in the carbon skeleton along with their influence on electrochemical performance. In this work, we aim to understand these properties in various GO materials derived from different graphitic sources. Both physiochemical and electrochemical characterization were employed to correlate the above-mentioned features and explore the effect of the location of the palladium nanoparticles (Pd NPs) on various GO supports for the hydrogen evolution reaction (HER). The interaction of the functional groups has a crucial role in the Pd dispersion and its electrochemical performance. Among the different GO samples, Pd supported on GO derived from graphene nanoplate (GNP), Pd/GO-GNP, exhibits superior HER performance; this could be attributed to the optimal balance among particle size, defect density, less in-plane functionalities, and higher electrochemical surface area. This study, thus, helps to identify the optimal conditions that lead to the best performance of Pd-loaded GO, contributing to the design of more effective HER electrocatalysts.

## 1. Introduction

The intellectual catalyst design approach is critical for developing advanced materials and accelerating various catalytic reactions, including those in industrial applications [1]. Among various techniques, particular importance is given to electrochemical water splitting reactions, which create clean, sustainable alternative energy to support the hydrogen economy [2,3]. Water splitting reactions proceed via two half-cell reactions: H^+^ ions are reduced at the cathode (hydrogen evolution reaction, HER), and the water is oxidized at the anode (oxygen evolution reaction, OER) [4]. While molecular hydrogen (H_2_) is not available naturally, the energy produced is clean and environmentally friendly, gaining interest in fuel cell technology and prompting researchers to necessitate its production on a large scale [5]. Moreover, H_2_ generated by the electrochemical approach appears to be greener and more sustainable [6]. Platinum (Pt) is considered the benchmark catalyst for HER in acidic media [7]; however, the limited abundance and high cost restrict its practical applications [8,9,10]. To reduce pressure on sole Pt, palladium (Pd), having a good affinity for hydrogen, can be an alternative [11,12]. However, the catalytic activity of Pd is lower compared to that of Pt since the Pd–hydrogen system tends to have an internal damage phenomenon due to hydrogen embrittlement that causes microstructural changes and cracking, thus reducing its mechanical integrity [13]. It requires inert support materials that can stabilize the Pd electrodes. In that direction, if the desired performance is achieved, efforts should still be made to reduce the metal content and maximize the HER performance to compensate for the cost to a techno-economic level and facilitate real-time applications. One of the strategies to address this issue is reducing the size of the nanoparticles (NPs) and then further decorating them on a high-surface-area substrate [14].

Graphene oxide (GO), a derivative of graphene, has various oxygen-containing functional groups, such as hydroxyl, epoxy, carbonyl, and carboxylic groups [15]. These functional groups act as binding sites for the reactants and allow transition metal ions to coordinate, making GO an excellent support candidate [16,17]. The utilization of Pd in its NPs form has the superior benefits of minimal usage while maintaining high catalytic efficiency when supported on GO [18]. This, in turn, makes the catalyst synthesis cost-effective. Electrochemical processes like HER benefit from binding Pd NPs on a GO support not only due to the uniform coverage of the metal nanoparticles (M-NPs) but also by improving stability [19,20]. GO supports have special advantages over other carbon supports for dispersing M-NPs. For example, typically existing carboxyl groups at the edges of GO sheets can efficiently serve as nucleation sites for Pd [21]. Hence, Pd NPs at higher concentrations are prone to attach at edge positions. If these groups distribute uniformly on the surface, their interaction can facilitate the anchoring of the Pd NPs, thereby providing a more homogeneous distribution. Likewise, epoxy groups lying on the basal plane of the GO sheet may also act as Pd binding sites [22]. GO supports also allow for controlling the degree of oxidation to maintain a balance between the sp^2^ hybridized carbon domains (related to conductivity and gas diffusion) and the functional groups introduced during oxidation (for dispersibility and functionalization). In general, the sp^2^ hybridized regions of GO are less functionalized and may not provide strong anchoring sites for Pd NPs, resulting in sparser Pd distribution in these areas. Benefitting from the different functional groups attached, the GO has distinct merits: it is capable of controlling the particle dispersion and morphology—creating more catalytic active sites—and improving the binding between Pd NPs and GO supports for better HER kinetics [23]. However, the actual roles of the functional group, their location (on the edges and/or basal plane), their influence on M-NPs’ dispersion, and catalytic performance are insufficiently explored, with more focus given to the M-NPs. Besides functional groups, other structural features like the interlayer spacing between the GO sheets and defects (distortion or vacancy) also demand careful consideration.

This work aims to investigate the physiochemical characteristics of a wide series of Pd-loaded GO catalysts resulting from various graphitic sources and explain their HER performance distinguishing it from our previous work, which focused mainly on the role of reactive functional groups and linker molecules in graphene oxide frameworks (GOF) for stabilizing Pd NPs, specifically in the context of the Suzuki–Miyaura reaction [24]. The present work deals with an electrochemical perspective, whereas the existing literature focuses more on reduced graphene oxide (rGO) rather than GO itself. The impact of functional groups and their influence on electrochemical performance, especially with GO, remains relatively underexplored. The results from this research will evidently show a clearer direction in designing effective HER electrocatalysts based on material characteristics and electrochemical behavior.

## 2. Materials and Methods

### 2.1. Materials

Four variations in GO materials were studied in the current research, among which three of them were reported in our previous publication [24]. The sample codes of the GO materials are summarized in Table 1. GO-GNP, GO-graphite45, and GO-graphite150 were prepared from graphite sources with different particle sizes. GO-commercial (dry powder, 50–100 mesh) was purchased from Layer One—Advanced Materials. Graphene nanoplatelets (particle size < 2 μm) were purchased from Strem Chemicals. Graphite (particle size > 45 µm, purity > 98%) was purchased from Wako Pure Chemical Industries. Graphite (particle size >150 μm) was supplied by Sigma Aldrich. The details of the synthetic protocols for GO materials are available in the previous publication [24].

Additionally, a graphene oxide framework (GOF) support was prepared from GO-GNP as a reference sample with the expectation of better dispersion and stabilization of Pd NPs in the confined spaces created by the covalently bonded linker molecules [25]. The GOF sample is referred to as GOF-GNP.

A Pd catalyst was synthesized by reducing PdCl_2_ in the presence of GO and GOF materials in toluene [25]. In this method, the reactive functional groups present on the support were exploited as reductant. For further details about the protocol, refer to our previous publication [24]. The catalyst where Pd was supported on a GO material is referred to as Pd/GO, and that for GOF-GNP is referred to as Pd@GOF-GNP.

Other reagents including diethyl ether (Wako Pure Chemical Industries, Osaka, Japan), palladium (II) chloride (PdCl_2_, Sigma Aldrich, St. Louis, MO, USA), phenyl diboronic acid (purity > 95.0%, Sigma Aldrich), hydrogen peroxide (35% aqueous solution, Tokyo Chemical Industry Co., Ltd., Tokyo, Japan), sulfuric acid (H_2_SO_4_) (Kanto Chemical Co., Inc., Tokyo, Japan), potassium permanganate (Kanto Chemical Co., Inc.), toluene (Kanto Chemical Co., Inc.), methanol (Kanto Chemical Co., Inc.), ethanol (Kanto Chemical Co., Inc.), propanol (Fujifilm Wako Pure Chemical Co., Osaka, Japan), 5% Nafion ^TM^ dispersion solution DE520 CS-type (Fujifilm Wako Pure Chemical Co.), and 0.3 μm and 0.05 μm alumina (Al_2_O_3_-Baikowski Co., Inc., Malakoff, TX, USA) were used without further purification.

### 2.2. Characterization

The FT-IR spectra were acquired using JASCO 6100 (JASCO, Tokyo, Japan) in the range of 4000–400 cm^–1^ with a resolution of 4 cm^–1^ by 16 scans. The sample powder was ground with dried KBr at a weight ratio of 1:50 and then pressed into a pellet. The Raman spectra of powder samples dispersed on a glass plate were recorded using a laser Raman spectrometer, NRS-4100 (JASCO), with an excitation wavelength of 532 nm and an exposure time of 25 s with 10 acquisitions.

The morphology, particle size, and particle size distribution of the Pd NPs were investigated by TEM using H-7650 (Hitachi, Tokyo, Japan) operating at an acceleration voltage of 100 kV. The samples were dispersed in ethanol with ultrasonication for 15 min, then dropped onto a carbon-coated copper grid and naturally dried overnight.

XPS was used to investigate the chemical composition and the oxidation state of individual elements. The spectra were recorded on Kratos AXIS Ultra DLD (Shimadzu, Kyoto, Japan) equipped with an Al-Kα anode. Powder samples were loaded onto a sample holder using double-sided adhesive copper tape. The survey spectrum was recorded with a 1 eV step size and a pass energy of 80 eV. Narrow scans were recorded with a 0.1 eV step size and a pass energy of 160 eV. The binding energies were calibrated using the C 1s peak of graphitic carbon at 284.6 eV. The sample powder was spread evenly on copper tape and measurement positions were determined by adjusting the stage position to the location where the C 1s peak of the sample exhibited the highest intensity. The spectra were analyzed using XPSpeak 4.1 software, with baseline correction performed using the Shirley method. The elemental composition was calculated by the area ratio of the detected peaks. Note that the Cu peak was not detected in any of the samples, ruling out the possibility that the observed C and oxygen-containing functional group originated from the copper tape.

### 2.3. Electrode Preparation

Electrochemical measurements were performed with an electrochemical workstation, HZ-7000, from Meiden Hokuto (Tokyo, Japan). The rotation speed was controlled with a rotating electrode device, HR series HR-500, connected to the workstation. A three-electrode cell was used, with a rotating disk electrode (RDE, diameter 5.0 mm and 0.196 cm^2^ surface area) as the working electrode, an Ag/AgCl electrode (3.0 M KCl) as the reference, and a graphite rod as the counter electrode. All the experiments were performed at room temperature. Before the electrochemical measurements, the RDE working electrode was polished with 0.3 μm Al_2_O_3_ to remove any deposited materials, followed by polishing with 0.05 μm Al_2_O_3_ to make a smooth surface. Then, it was sonicated in water for 30 s using an ultrasonic bath to remove any deposited particles from the surface. A total of 10 mg of catalyst powder was initially dispersed in a mixture containing 1.50 mL of H_2_O, Milli-Q water (ultrapure water produced by a Milli-Q purification system, Millipore, Burlington, MA, USA), 480 μL propanol, and 20 μL Nafion ^TM^ binder (5.0 wt%). The solution was ultrasonicated with a bath sonicator for 45 min to attain a uniform dispersion, followed by 5 min of probe sonication. Finally, 10 μL of the prepared ink was drop cast on the RDE surface, covered with a vial, and naturally dried at room temperature. The catalyst loading was set to 0.255 mg cm^−2^.

### 2.4. Electrochemical Measurements

Linear sweep voltammetry (LSV) polarization curves were measured using 0.5 M H_2_SO_4_ as the electrolyte at a scan rate of 5 mV s^−1^ and with a rotation speed of 1600 rpm to study the HER performance of the catalysts. During the measurement, the electrolyte was continuously purged with nitrogen gas using a surface bubbler to prevent atmospheric oxygen. The Tafel slope was obtained from the LSV polarization curve using the logarithmic relationship between overpotential and current density. The double-layer capacitance (C_dl_) values were calculated by performing cyclic voltammetry (CV) at different scan rates ranging between 10 and 100 mV s^−1^ in a non-Faradaic region and by plotting the ∆j = (J*_a_* − J*_c_*) vs. scan rate, where J*_a_* and J*_c_* are the current measured at the anodic and cathodic regions, respectively. The electrochemical active surface area (ECSA) was calculated using the following relationship [26],

ECSA = Geometrical Surface area × C_dl_/C_s_

where C_s_ is the specific capacitance value (40 μF cm^−2^) [27] and the geometric surface area is 0.196 cm^2^.

During electrochemical measurements, the rotation of the working electrode and the evolved bubbles can cause the coated catalyst to peel off. Therefore, the durability test was performed on a 1 × 1 cm^2^ carbon paper substrate as the working electrode, where the catalyst loading normalized concerning the electrode surface area remains consistent.

The potential specified in this work is referenced to the reversible hydrogen electrode (RHE).

**E** (vs. RHE) = **E** (vs. Ag/AgCl) + 0.059 × pH + 0.197 V

Electrochemical impedance spectroscopy (EIS) measurements were performed with an electrochemical workstation, HZ-Pro S4, from Meiden Hokuto. The measurements were carried out at an open circuit potential in a frequency range from 20 kHz to 0.01 Hz with an AC amplitude of 10 mV. All the polarization curves shown are without i-R compensation.

## 3. Results and Discussion

### 3.1. Structural Characterizations

**Figure 1:**
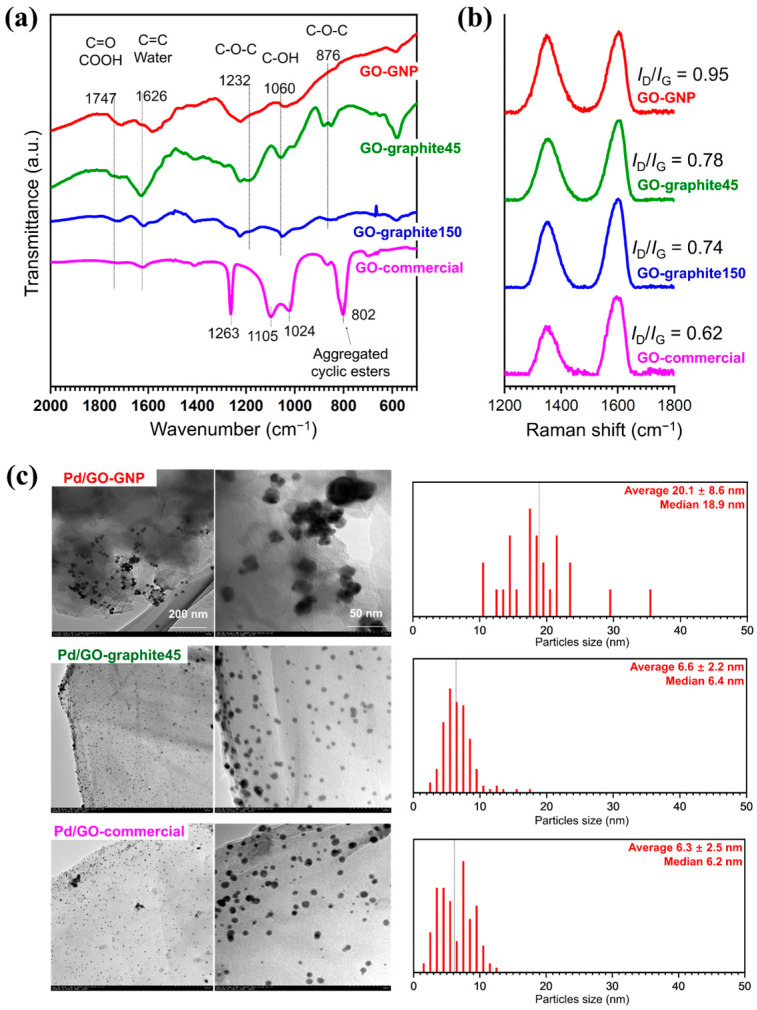
Characterization of the GO supports and the relevant Pd catalysts: (**a**) FT-IR and (**b**) Raman spectra of the supports; (**c**) TEM images for the supported Pd catalysts in two different magnifications. The particle size distribution of Pd NPs is shown on the right side of each image. The particle size was determined by analyzing 200 randomly selected particles at fixed magnification using ImageJ software [28]. The IR and Raman spectra as well as the TEM images for GO-GNP, GO-graphite45, GO-commercial, and the relevant Pd catalysts are from our previous publication [24].

All the GO and Pd/GO samples other than Pd/GO-graphite150 and their support were already characterized in the previous publication [24] so their differences are only briefly explained here. The IR and Raman spectra of the GO samples are shown in Figure 1a,b. The GO samples prepared in-house (GO-GNP, GO-graphite45, and GO-graphite150) had in-plane functional groups such as epoxy (C-O-C) and hydroxyl (C-OH), while GO-commercial possessed a smaller number of in-plane functional groups but many aggregated cyclic esters. GO-GNP was the most defective due to the presence of many edges and GO-commercial was the least defective due to the largest lateral size of the sheets and the least in-plane functionalization.

Figure 1c shows the TEM images of the prepared Pd/GO catalysts together with the particle size distribution of Pd NPs calculated from the image. Pd/GO-GNP showed the largest particle size (average diameter = 20.1 ± 8.6 nm) among the catalyst samples. The reason for the smaller Pd NPs of Pd/GO-graphite45 (6.61 ± 2.2 nm) and Pd/GO-commercial (6.31 ± 2.5 nm) is probably the formation of Pd NPs between stacked GO sheets. GO-GNP had almost no such space due to the severe exfoliation, which resulted in the formation of significantly coarse Pd NPs.

The XPS elemental analysis results (Table 2) show that GO-GNP was less functionalized compared to the other GO supports as it exhibited ca. 3–4 wt% lower oxygen content. This tendency is maintained even after Pd loading. The higher Pd loading of Pd/GO-GNP and Pd/GO-commercial is attributed to the presence of rough Pd particles deposited on the outer surface [24].

### 3.2. Electrochemical Performance

**Figure 2:**
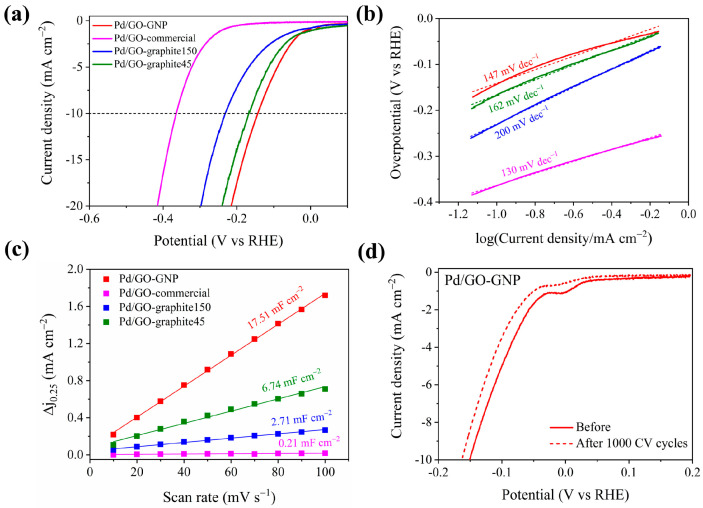
Electrocatalytic activity of synthesized catalysts: (**a**) HER polarization curves measured in 0.5 M H_2_SO_4_ at 1600 rpm rotation, its corresponding (**b**) Tafel plots, (**c**) C_dl_ plot obtained from the CV (Figure S3), (**d**) durability test: polarization curves obtained before and after 1000 CV cycles at 5 mV s^−1^ scan rate.

The HER performances of Pd/GO catalysts were evaluated using the LSV technique with a 1600 rpm rotation speed. The catalytic current at 10 mA cm^−2^ was fixed as the benchmark for comparing the performance of different catalysts. Among all the catalysts, Pd/GO-GNP showed the best catalytic performance for HER with a lower overpotential (143 mV@10mA cm^−2^) than the others, Figure 2a. The activity trend followed in the order of Pd/GO-GNP > Pd/GO-graphite45 > Pd/GO-graphite150 > Pd/GO-commercial. Control experiments were performed using GOs without the Pd loading under identical conditions (Figure S1). The resultant LSV curves confirmed that no significant activity was brought by GO alone, suggesting that GO itself is less conductive. The improved activity may arise from the combined benefits of Pd NPs and GO support. It can be anticipated that when supported on GO, Pd/GO catalysts exhibit enhanced catalytic performance because of better Pd dispersion and electronic interactions with GO and by offering high surface-to-volume ratios, with an increased number of catalytic sites available for reactions.

The Tafel slopes obtained from the polarization curves were used to analyze the intrinsic nature of the catalysts. The Pd/GO-GNP had a lower Tafel value (147 mV dec^−1^) compared to Pd/GO-graphite45 (162 mV dec^−1^) and Pd/GO-graphite150 (200 mV dec^−1^), which indicates its faster HER kinetics, Figure 2b. This, in turn, suggests that electron transfer is more facilitated in Pd/GO-GNP than in other catalysts. Note: The Tafel slope specifically measures the rate-determining step’s sensitivity to the overpotential and not the overall activity or current density. The Pd/GO commercial catalyst has a low Tafel slope with a lower current density, indicating less overall H_2_ production despite favorable reaction kinetics. This may be accounted for by its limited number of exposed active sites. Compared to other forms of GO, GO-GNP generally contains fewer in-plane defects, thereby retaining higher structural integrity, allowing the electron pathways to remain less disrupted and electron transfer to occur more smoothly and efficiently across the material. One possibility is that the interactions between Pd NPs and the GNP surface create an electronically favorable environment, modifying the electronic structure of Pd and thus enhancing its catalytic property to facilitate faster electron transfer. To validate this claim, the EIS technique was used to probe the interfacial electron transfer kinetics of the catalysts. The impedance spectra were represented in a Nyquist plot, Figure S2a, and fitted with a Randles circuit, R(QR), shown schematically in Figure S2b. The elements used for fitting and its summarized results are tabulated in Table S1. The solution resistance (R_s_) remains the same for all the catalysts. The charge transfer resistance (R_ct_) can be easily visualized in Figure S2a. The smaller the semicircle, the smaller the R_ct_, and the higher the charge transfer. In other words, R_ct_ is inversely proportional to the current density. Pd/GO-GNP exhibited the lowest R_ct_ value, confirming the highest conductivity among the tested catalysts. The results are thus consistent with the Tafel plot. The larger particle size of GO and stacking of GO sheets together hinder the electron transfer process in all the Pd-loaded GO catalysts except for Pd/GO-GNP.

The C_dl_ was determined to shed deeper insight into improved performance, giving better knowledge about electrochemically active sites of samples. Figure 2c shows the C_dl_ values obtained by performing CV at various scan rates (Figure S3). Among them, Pd/GO-GNP had the largest C_dl_ value of 17.51 mF cm^−2^ compared with Pd/GO-commercial (0.21 mF cm^−2^), Pd/GO-graphite45 (6.74 mF cm^−2^), and Pd/GO-graphite150 (2.71 mF cm^−2^). Since electrochemical surface area (ECSA) is proportional to the C_dl_ values, the ECSA values were calculated and are displayed in Table S2. In general, the presence of functional groups on GO can facilitate the uniform dispersion and anchoring of Pd NPs with many accessible active sites for catalytic reaction by acting as nucleation sites. It should be highlighted that Pd loading on Pd/GO-GNP, as evidenced by the TEM image, is onto the surface of GO-GNPs, unlike any other type of GO material wherein Pd NPs are stacked between the layers. In addition, the size of the GO-GNP is noteworthy as it is smaller—but higher in surface area—compared to other GOs, making it more accessible for electrochemical reactions. Since the surface topology can influence the HER performance, the obtained LSV results were normalized by the ECSA value to determine the actual source of activity. The obtained ECSA-normalized HER plot (Figure S4) revealed that the HER activity trend is different while normalized with ECSA values. The HER activity followed the order: Pd/GO-commercial > Pd/GO-graphite150 > Pd/GO-GNP > Pd/GO-graphite45. These observations support the fact that the electrochemical activity obtained is mainly due to the enhanced electrochemical surface area of the catalyst, for which one cannot rule out the role of functional groups that indirectly contribute to achieving a high electrochemical surface area.

Summarizing all the electrochemical measurements, it is reasonable to conclude that poorer functionality generates faster charge transfer kinetics; however, to validate this hypothesis, we further expanded our studies to evaluate the electrochemical performance of two types of GO-GNP, i.e., Pd/GNP and Pd@GOF-GNP. The extent of functionalization in GOF is usually higher than in GO due to the introduction of cross-linkers or other functional groups, providing a porous or extended network. As expected from the electrochemical HER results (Figure S5), a better activity of Pd/GO-GNP compared to Pd/GNP and Pd@GOF-GNP is determined. Although Pd@GOF-GNP has well-dispersed Pd NPs, their confinement between the GO layers makes it less accessible for the catalytic reaction. This is further evident from the obtained C_dl_ values displayed in Figure S6. The lower C_dl_ value of Pd@GOF-GNP compared to Pd@GO-GNP could be attributed to its fewer exposed electrochemically active sites. CV measurements (Figure S7) were performed to identify the distinct role of Pd NPs. The palladium peak at +0.1 V vs. RHE reflects that Pd/GO-GNP has more exposed active sites than Pd/GO-graphite45 and other catalysts. This finding correlates well with the TEM results. Altogether, it could be concluded that the Pd NPs’ distribution on or in between the GO layers has a critical role in determining HER performance, suggesting that tailoring the functional groups for more exposure to the active surface might show better electrochemical performance.

**Figure 3:**
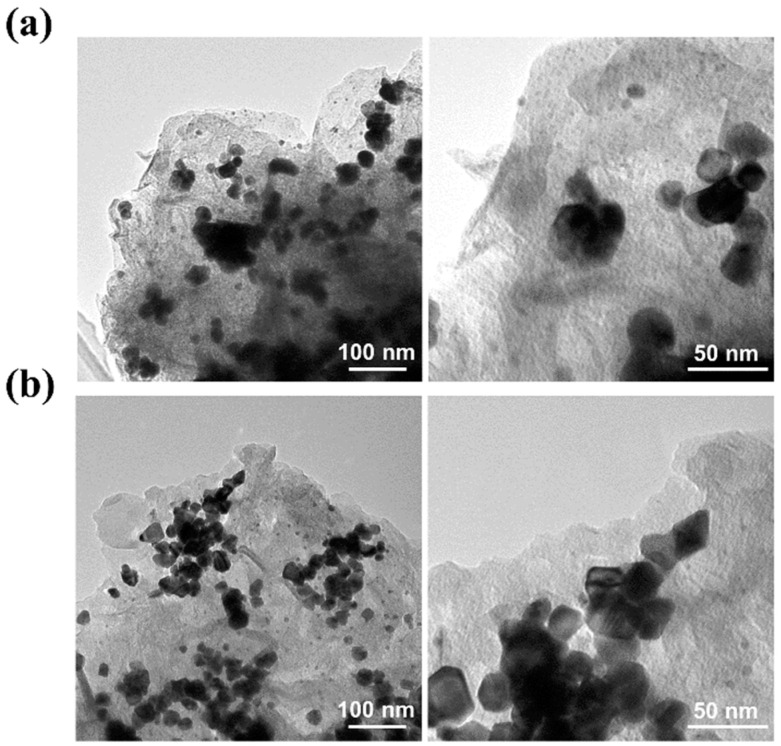
TEM images for the Pd/GO-GNP catalysts (**a**) before and (**b**) after 1000 CV cycles.

Stability is an important criterion when proposing an electrocatalyst for practical applications. To analyze it, the HER polarization curves were measured before and after 1000 CV cycles. Figure 2d shows the HER polarization curves of Pd/GO-GNP measured before the 1st CV cycle (solid line) and after (dotted line) the 1000th CV cycle. The result reveals only an increase of 13 mV in overpotential, which implies reasonable stability. Such activity loss could be related to bubble formation that may lead to the catalyst leaching during the HER process. To rule this out, the catalyst with the same catalyst loading was coated on carbon paper, and the stability test was carried out. Figure S8 shows almost no change in the polarization curve, thus confirming excellent durability. The interaction of the Pd NPs with GO’s functional groups prevents their sintering or aggregation during catalytic reactions, therefore remaining at a high surface area over time. No characteristic changes were observed in Pd NPs, which were further corroborated by the TEM images shown in Figure 3, displaying Pd/GO-GNP as a better electrocatalyst. XRD analyses were performed before and after the chronoamperometry test, Figure S9a–c. The results reveal that a negative shift is observed in the Pd peaks, suggesting the formation of Pd-hydride species [29,30].

## 4. Conclusions

In summary, we state the critical role of functional groups and other structural features of graphitic-material-supported catalysts—Pd/GO—in electrochemical HER performance. Some key findings of this study are summarized as follows: 1. GO support in-plane functionalities have a significant role in Pd dispersion and anchoring. While the surfaces of Pd NPs are exposed on the lower-functionality GO-GNP support, for other GOs—although Pd NPs are small and homogeneous—the Pd NPs are entrapped between the layers, making them less accessible. 2. Apparently, the lateral dimensions of GO are very important in deciding its catalytic performance. While small GO/GNP sheets form a well-connected network with a high electrochemically active surface area for fast electron transfer, larger sheets in GO-graphite45 and GO-graphite150 hinder this. The Pd/GO-GNP, having the highest activity of 143 mV@10mA cm^−2^, benefits primarily from the higher electrochemical surface area together with optimal particle size, less in-plane functionalities, and defect density, which ultimately contributes to achieving it. The present work outlines the possibility of using GO supports in the rational design of HER electrocatalysts by tailoring their physiochemical properties.

## Figures and Tables

**Table 1:** Sample codes for GO samples.

Carbon Material	Sample Code
N.A. *^a^*	GO-commercial
Graphite (>45 μm)	GO-graphite45
Graphene nanoplatelets (<2 μm)	GO-GNP
Graphite (>150 μm)	GO-graphite150

*^a^* The GO was purchased from a commercial source.

**Table 2:** Elemental composition determined by XPS.

	C (wt%)	O (wt%)	Cl (wt%)	Pd (wt%)
GO-GNP ^a^	65.5	34.5		
GO-graphite45 ^a^	61.4	38.6		
GO-graphite150	55.9	44.1		
GO-commercial ^a^	62.1	37.9		
Pd/GO-GNP ^a^	49.7	25.1	8.6	16.7 (16.0/0.6) ^b^
Pd/GO-graphite45 ^a^	57.1	29.3	4.0	9.7 (8.9/0.7) ^b^
Pd/GO-graphite150	45.8	39.6	3.33	11.3 (7.5/3.8) ^b^
Pd/GO-commercial ^a^	44.1	30.1	8.4	17.5 (15.7/1.7) ^b^

^a^ These data are from our previous publication [24]. ^b^ The content of Pd(0) and Pd(II) are shown in parentheses as (Pd(0)/Pd(II)).

## Data Availability

The original contributions presented in the study are included in the article/supplementary material, further inquiries can be directed to the corresponding authors.

## Supplementary Materials

The following supporting information can be downloaded at: https://www.mdpi.com/article/10.3390/ma17215296/s1. Electrochemical measurements such as LSV, Nyquist plot, C_dl_, CV measurements, and stability test are given in Figure S1 to S9 along with Table S1, showing the EIS results, and Table S2 displaying the ECSA values derived from C_dl_.
