# Supplementary material for: Structure-Driven Performance Enhancement in Palladium–Graphene Oxide Catalysts for Electrochemical Hydrogen Evolution
Source: Materials (Basel). 2024 Oct 31;17(21):5296. doi: 10.3390/ma17215296 (PMC11547229; doi:10.3390/ma17215296)
Supplement: Supplementary file 1 [file materials-17-05296-s001.zip › materials-3242545-supplementary.pdf]

SUPPORTING INFORMATION

Structure-Driven Performance Enhancement in Palladium-Graphene Oxide Catalysts for Electrochemical Hydrogen Evolution

Krishnamoorthy Sathiyan, * *Ce Gao, Toru Wada, Poulami Mukherjee, Kalaivani Seenivasan, Toshiaki Taniike**

Graduate School of Advanced Science and Technology, Japan Advanced Institute of Science and Technology, 1-1 Asahidai, Nomi, Ishikawa, 923-1292, Japan

* *Corresponding authors*

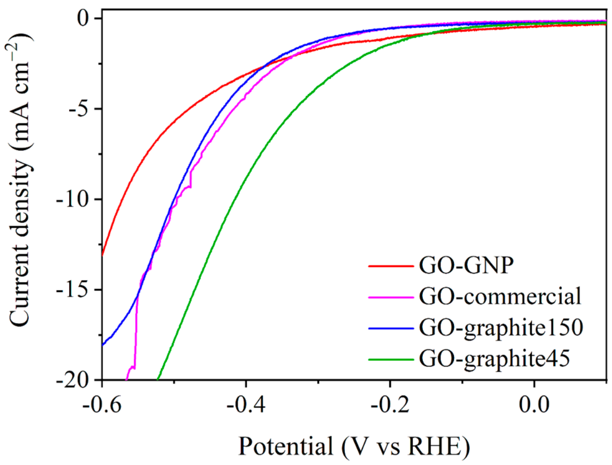

Figure S1. HER polarization curves of various GO samples (without Pd) measured in 0.5 M H_2SO_4 at 1600 rpm rotation.

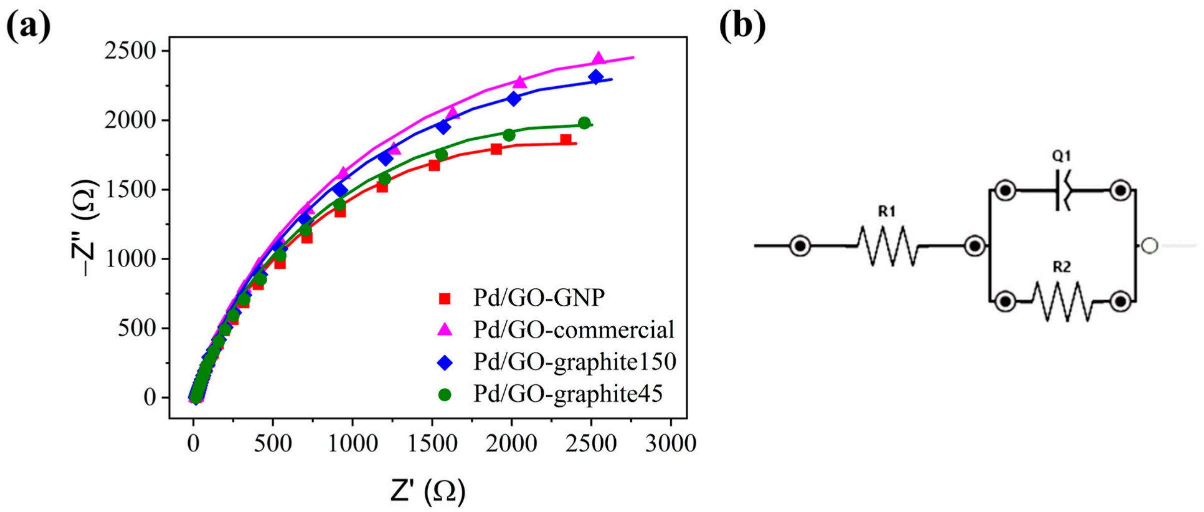

Figure S2. (a) Nyquist plot of all the catalysts, and (b) the equivalent circuit model used for the fitting.

Table S1. The fitted electrochemical results of the EIS parameters for different catalysts.

Electrocatalysts	R_s	Q	R_{ct}
Pd/GO-GNP	16.56 ± 0.81	0.8636	4555 ± 4
Pd/GO-commercial	16.57 ± 0.61	0.8657	6104 ± 3
Pd/GO-graphite150	16.76 ± 0.82	0.8637	5720 ± 4
Pd/GO-graphite45	16.46 ± 0.81	0.8639	4880 ± 4

$\mathbf{R_s}$ is the uncompensated solution resistance.

Q is the constant phase element (CPE) associated with the electrochemical double-layer capacitance at the catalyst | electrolyte interface.

$\mathbf{R_{ct}}$ is the charge transfer resistance between the interface of the electrode and electrolyte.

Note: The impedance measurements were performed using a 1 x 1 cm^2 carbon paper substrate as the working electrode with the same catalyst loading normalized relative to the electrode surface area. The error mentioned in the table represents relative standard error.

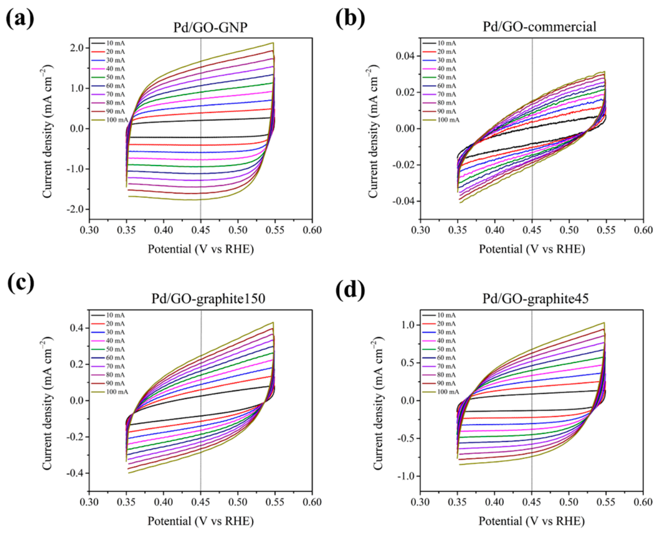

Figure S3. Cyclic voltammogram curves measurements in the non-faradic region to obtain the double-layer capacitance (C_{dl}).

Table S2. The obtained C_{dl} value and its derived ECSA values for different catalysts.

**Electrocatalysts**	**C_{dl} (mF/cm^2)**	**ECSA (cm^2)**
Pd/GO-GNP	17.51 ± 0.52	85.82
Pd/GO-commercial	0.21 ± 0.04	1.02
Pd/GO-graphite150	2.71 ± 0.25	13.28
Pd/GO-graphite45	6.74 ± 0.21	33.01

The error represents the standard deviation.

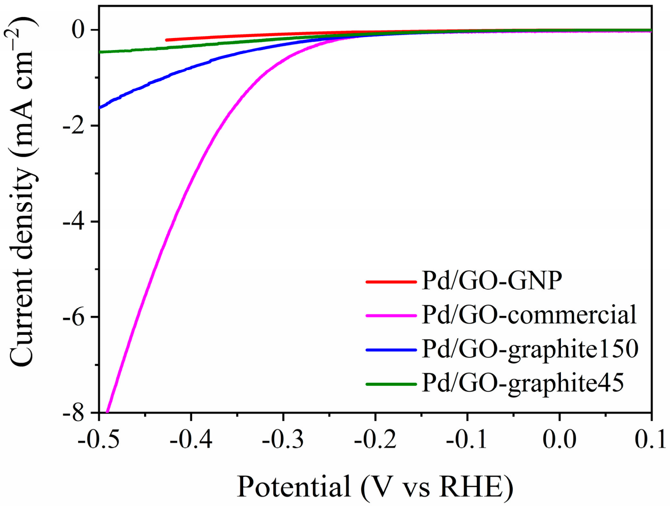

Figure S4. A plot of HER polarization curves normalized by ECSA.

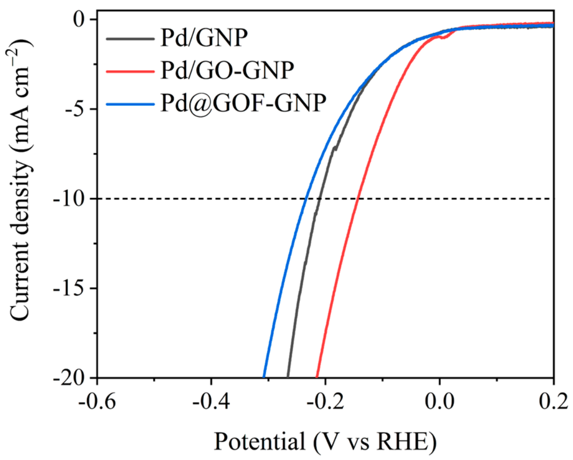

Figure S5. HER polarization curves of Pd with GNP material: Pd/GNP (without GO oxidation), Pd/GO-GNP (after GO oxidation), and Pd@GOF-GNP (forming graphene oxide framework) measured in 0.5 M H_2SO_4 at 1600 rpm rotation.

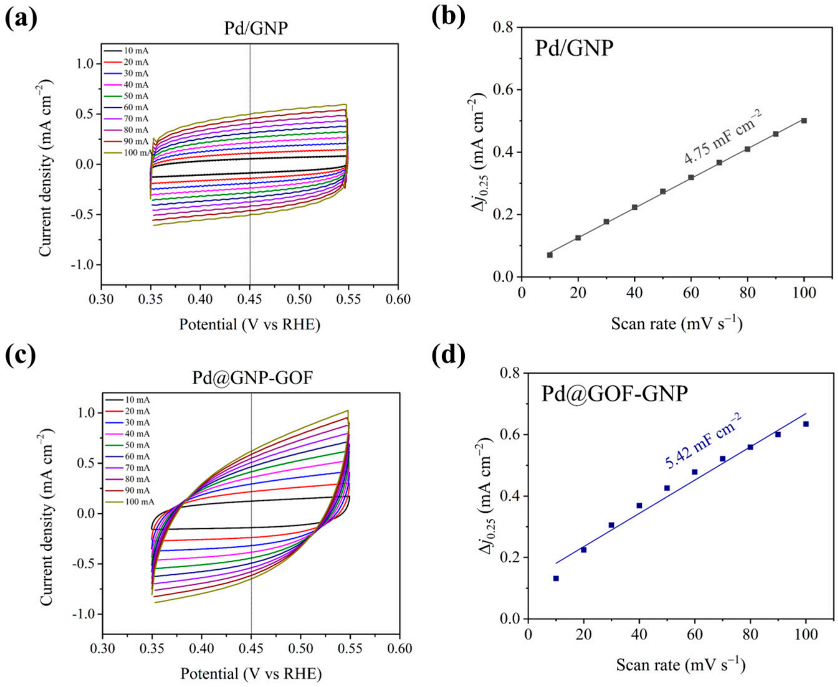

Figure S6. (a, c) CV in a non-faradaic region, and (b, d) their corresponding C_{dl} values.

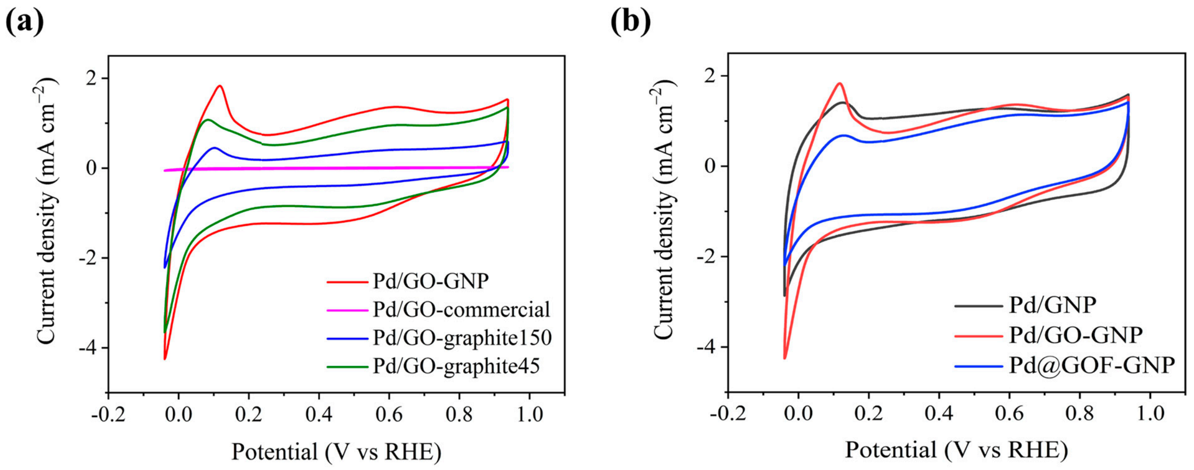

Figure S7. Cyclic voltammogram of various Pd-GO catalysts measured in 0.5 M H_2SO_4 solution at a scan rate of 50 mV s^{-1}.

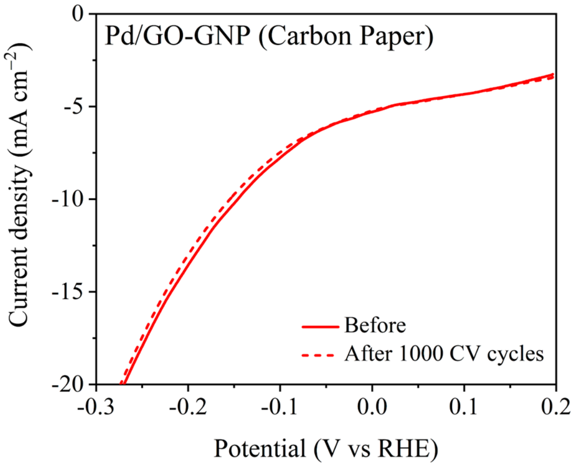

Figure S8. Durability test: HER polarization curves obtained before and after 1000 CV cycles at 5 mV s^{-1} (Pd/GO-GNP catalyst coated on carbon paper substrate).

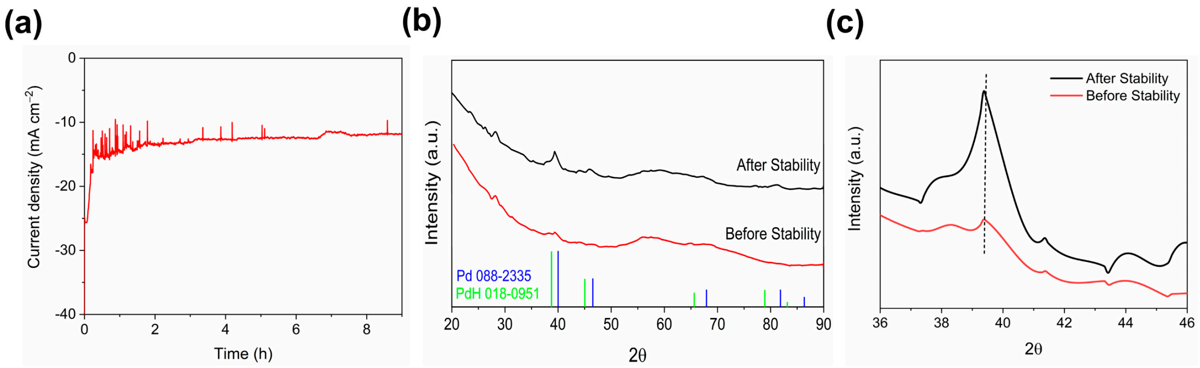

Figure S9. (a) Chronoamperometry measurement performed at 10 mA cm^{-2} current for Pd/GO-GNP catalyst coated on carbon paper, (b) XRD pattern of Pd/GO-GNP catalyst before and after stability test, and (c) its zoomed-in image showing the formation of Pd hydride NPs.
